# The context-dependent roles of PPAR-γ in adipocyte differentiation and obesity: a master regulator with dual functions

**DOI:** 10.3389/fnut.2026.1820776

**Published:** 2026-05-28

**Authors:** Xiaoli Hou, Yiqiu Chen, Weixia Dong, Xiao Li, Shaoping Ji

**Affiliations:** 1 Center for Molecular Medicine, Zhengzhou Health College, Zhengzhou, Henan, China; 2 Department of Gastroenterology, Huaihe Hospital of Henan University, Kaifeng, Henan, China; 3 Department of Biochemistry and Molecular Biology, Medical School, Henan University, Kaifeng, Henan, China

**Keywords:** chronic inflammation, insulin resistance, metabolic syndrome, obesity, PPAR-γ signaling, therapeutic target

## Abstract

Obesity has become a global public health crisis. It is characterized by pathological proliferation of adipose tissue resulting from an energy metabolism imbalance. Adipocyte differentiation is the core process driving this proliferation and is tightly regulated by a complex molecular network. Peroxisome proliferator-activated receptor gamma (PPAR-γ) is the primary regulator of adipocyte differentiation. It also plays a key regulatory role in lipid and glucose metabolism as well as inflammatory responses. Its dysregulation is closely associated with the onset and progression of obesity. This review goes beyond a descriptive summary of PPAR-γ’s role in adipogenesis and instead focuses on the context-dependent roles of this factor in the pathophysiology of obesity. This article maps the upstream and downstream molecular networks regulating PPAR-γ activity. It also analyzes the dual and even opposing functions that PPAR-γ exhibits under different physiological and pathological conditions. By integrating cutting-edge research advances, this review further elucidates the tissue-specific mechanisms of PPAR-γ and evaluates the therapeutic potential of targeting this pathway in obesity interventions. This review aims to provide a theoretical framework for understanding the mechanisms by which PPAR-γ dysfunction contributes to the development of obesity. It offers a reference for the development of precision treatment strategies for obesity and related metabolic diseases.

## 1 Introduction

In recent years, the sharp rise in global obesity prevalence has become a serious public health challenge (1–7). Obesity is not merely the result of an energy imbalance but involves systemic dysregulation of adipose tissue structure and function (8–11). It is often accompanied by low-grade systemic inflammation and significantly increases the risk of metabolic diseases such as type 2 diabetes and cardiovascular disease (12–14). Traditional obesity research has primarily focused on the energy storage function of white adipose tissue and its pathological expansion (15, 16). However, the discovery of thermogenic activity in brown and beige adipose tissues, as well as the plastic differentiation of “pink fat” in mammary tissue (17, 18), has revealed the critical role of adipocyte trans-differentiation and functional adaptation in metabolic regulation. Against this backdrop, peroxisome proliferator-activated receptor gamma (PPAR-γ) is widely recognized as a central transcription factor in adipogenesis (19, 20). However, its regulatory role exhibits significant variability across different adipocyte types, metabolic states, and tissue microenvironments, with even conflicting functional reports. This ambiguity reflects the current controversies and knowledge gaps in the field.

Much of the existing literature emphasizes the positive role of PPAR-γ in promoting adipocyte differentiation and maintaining insulin sensitivity (21–24). However, its role in the progression of obesity is by no means singular. Overexpression of PPAR-γ promotes lipid accumulation and tissue expansion in white adipose tissue. In brown/beige adipose tissue, however, it enhances thermogenesis and energy expenditure (25–27). More importantly, emerging evidence suggests that under specific pathological conditions, PPAR-γ is involved in pro-inflammatory and fibrotic processes (28–31). This challenges the traditional view of PPAR-γ as a purely metabolic-improving factor. Furthermore, although PPAR-γ ligands such as thiazolidinediones (TZDs) have demonstrated clear efficacy in improving insulin resistance, their clinical application is limited by side effects such as weight gain and edema (32–35). This reflects that the tissue specificity of the PPAR-γ signaling pathway, the complexity of its regulatory networks, and its context-dependence have not been fully elucidated.

In response to the aforementioned research gaps, this review focuses on the multidimensional and bidirectional roles of PPAR-γ in various adipocyte differentiation pathways, the development of obesity, and metabolic inflammatory networks. It analyzes conflicting conclusions and mechanistic controversies in current research. The aim is to establish an integrative framework that provides a theoretical basis for targeted interventions in obesity treatment.

## 2 Expression and regulation of PPAR-γ

The human PPAR-γ gene is located in the 3p25.2 region and primarily encodes two protein subtypes: PPAR-γ1 and PPAR-γ2 (36). PPAR-γ is a member of the nuclear receptor superfamily (37–42). It is involved in the regulation of lipid metabolism, glucose homeostasis (43–49), inflammatory responses (50–56) and fibrogenesis (39, 57). However, PPAR-γ exhibits a marked preference for expression in adipose tissue, with the highest levels observed in white fat (58–60). This establishes its role as the “master regulator” of adipocyte differentiation and function.

Notably, this expression pattern is not static. Under metabolic stress conditions such as obesity, insulin resistance, or inflammation, PPAR-γ expression levels and activity undergo dynamic changes (61–63). Furthermore, significant differences exist between subcutaneous and visceral adipose tissues (64–68). This highlights the highly context-dependent nature of its regulation.

Structurally, PPAR-γ consists of the N-terminal ligand-independent activation domain 1 (AF1), the DNA-binding domain (DBD), the hinge region, and the C-terminal ligand-binding domain (LBD) and ligand-dependent activation domain 2 (AF2) (36). The large and flexible ligand-binding pocket within the LBD can accommodate a variety of endogenous and exogenous ligands (69, 70). Although this characteristic broadens its signaling scope, it also increases the risk of non-specific regulation. Taking TZDs as an example, these potent synthetic ligands significantly activate PPAR-γ and improve insulin sensitivity. However, they also cause side effects such as weight gain due to excessive activation of fat storage pathways (71, 72). This dilemma raises a critical question: Is there a tissue-specific or functionally selective ligand-binding mode capable of separating the metabolic benefits of PPAR-γ from its adverse effects?

At the transcriptional regulation level, PPAR-γ typically forms heterodimers with retinoic acid X receptor (RXR), thereby binding to PPREs and initiating the transcription of target genes (73). The traditional view emphasizes the central role of positive feedback loops between PPAR-γ and the C/EBP family (particularly C/EBPα/β) in adipocyte differentiation (74). However, recent studies increasingly reveal that the transcriptional output of PPAR-γ is highly dependent on the dynamic recruitment of coactivators (such as PGC-1α) and co-repressors. In turn, the expression and activity of these cofactors are precisely regulated by nutritional status, inflammatory signals, and epigenetic modifications (75–77). For example, under conditions of energy excess, the activation of inflammatory pathways such as NF-κB and STAT alters the gene expression profiles of PPAR-γ or its co-regulators. Consequently, its function shifts from metabolic regulation to pro-inflammatory effects (71, 78). Whether this shift constitutes a key mechanism underlying obesity-associated chronic inflammation remains to be elucidated.

Notably, its activation can suppress certain inflammatory mediators and exhibits anti-fibrotic potential (79), However, in specific pathological contexts (such as adipose tissue fibrosis and non-alcoholic steatohepatitis), PPAR-γ signaling conversely promotes inflammatory and fibrotic processes (30, 31, 80). This seemingly contradictory double-edged sword effect highlights the context-dependence of its regulatory network and suggests that the current research paradigm based on “global activation or inhibition” is overly simplistic.

In summary, the expression and regulation of PPAR-γ constitute a multi-layered dynamic network. Its functional output depends on multiple factors, including tissue type, metabolic environment, ligand specificity, and co-regulatory factors. Future research must move beyond static descriptions of its structure and signaling pathways. Instead, it should focus on the plasticity of its regulation and the mechanisms underlying functional transitions under both physiological and pathological conditions.

## 3 Core molecular mechanisms of PPAR-γ in regulating adipocyte differentiation

### 3.1 Programmed expression of PPAR-γ in adipocyte differentiation

The differentiation of adipocytes is critical for the formation and proliferation of white adipose tissue (59). However, the molecular turning point at which this process shifts from adaptive growth to pathological proliferation in the context of obesity remains unclear. The regulatory role of PPAR-γ in this transition is one of the central points of contention in current research.

**FIGURE 1:**
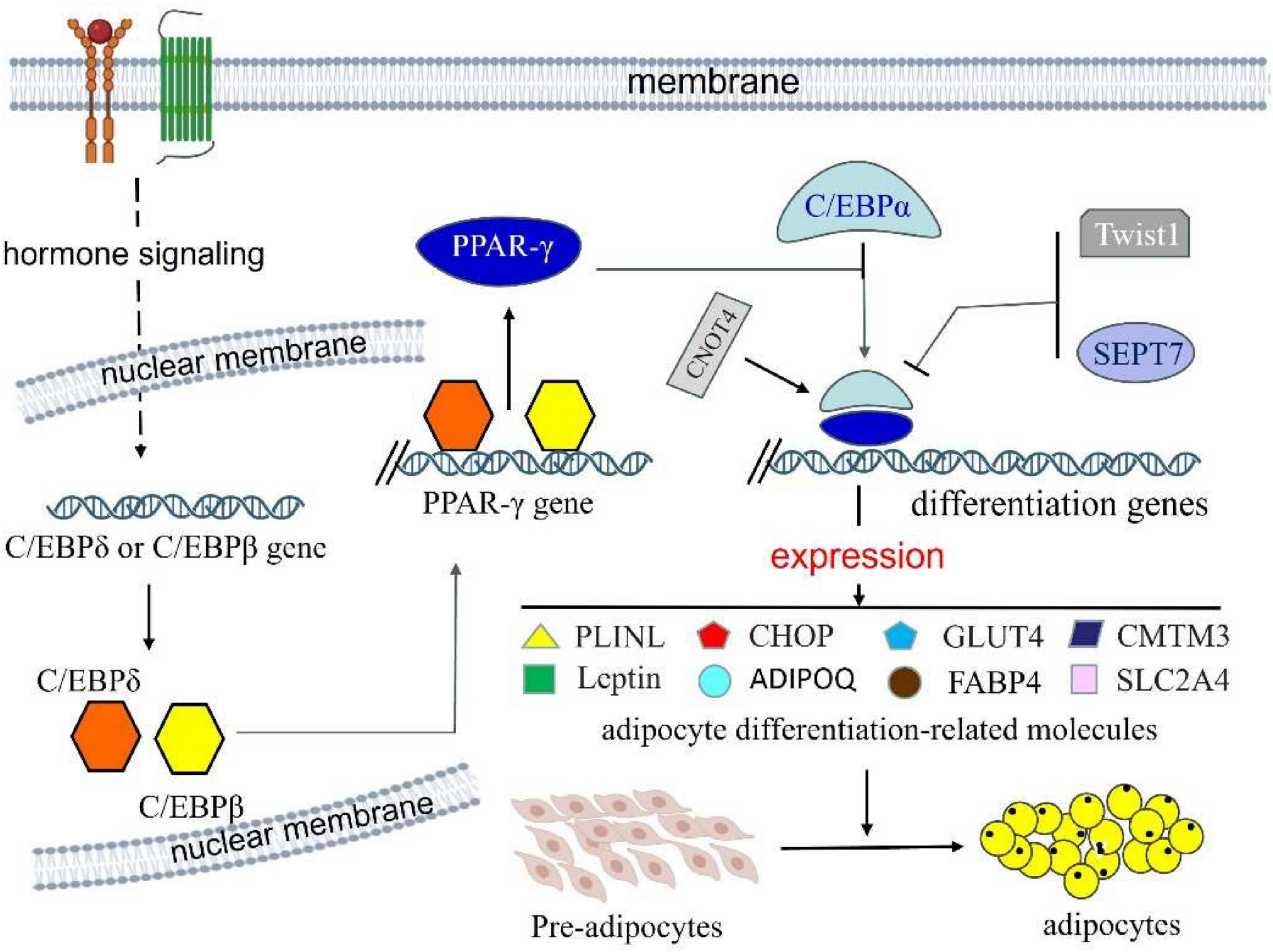
Schematic representation of pre-adipocyte differentiation mediated by PPAR-γ in multiple steps. At the initial stage, membrane receptors on preadipocytes are stimulated by extracellular hormonal signals such as insulin and catecholamines. These signals are transmitted into the nucleus, leading to the expression of the adipogenic transcription factors C/EBPβ and C/EBPδ. These two factors bind to the CCAAT box within the PPAR-γ promoter region, thereby inducing PPAR-γ expression. Subsequently, PPAR-γ acts as a master transcription factor, forming a heterodimer with C/EBPα and binding to the promoter regions of adipocyte differentiation–related genes (such as FABP4 and ADIPOQ), which promotes the expression of genes involved in lipid synthesis and storage. This regulatory network is precisely controlled at multiple levels: for example, CNOT4 promotes adipose tissue growth by enhancing PPAR-γ transcriptional activity; Twist1, on the other hand, limits adipogenesis through a triple inhibitory mechanism—binding to promoters, blocking protein activity, and competing with RXRα; meanwhile, SEPT7 attenuates adipogenesis and promotes lipolysis by targeting PPAR-γ and C/EBPα.

The classical adipogenesis model describes a cascade of events initiated by the activation of C/EBPβ/δ. This subsequently induces PPAR-γ expression, forming a positive feedback loop with C/EBPα (19, 81, 82). This loop drives the expression of functional genes such as FABP4 and adiponectin (26, 83–85), promoting lipid storage and maintaining insulin sensitivity (86, 87) (Figure 1). However, this model fails to thoroughly explain a key paradox: under conditions of nutritional excess, the same pathway maintains metabolic homeostasis, whereas in the context of inflammation and insulin resistance, it drives adipose tissue dysfunction.

The key to resolving this issue may lie in the “dose-response” and “context-dependent” characteristics of PPAR-γ activity. Studies have shown that the transcriptional activity of PPAR-γ is not simply “on” or “off.” The activation of its downstream target genes is finely tuned by synergistic regulatory networks (88–92). For example, PPAR-γ synergistically promotes lipid synthesis with SREBP-1c (93). Similarly, a variety of regulatory factors have been identified, including the activator CNOT4, the repressor Twist1, and the lipolysis-promoting factor SEPT7, which together constitute a complex “fine-tuning” system (59, 74, 94).

These regulators are differentially expressed in various types of adipose tissue (such as visceral fat and subcutaneous fat) and under different metabolic conditions (95). This determines whether PPAR-γ signaling drives healthy fat storage or pathological adipose tissue remodeling and inflammation.

The transition from physiological to pathological state essentially reflects the functional remodeling of the PPAR-γ regulatory network under metabolic stress. Current research still lacks a dynamic analysis of this transition process. Therefore, future studies urgently need to shift from static cataloging of “pathway components” to constructing “dynamic regulatory network models.” This will reveal how nutritional, hormonal, and inflammatory signals integrate and reshape the functional output of PPAR-γ, thereby elucidating the molecular basis of obesity-related adipose tissue dysfunction.

### 3.2 Epigenetic and post-translational modification regulation of the PPAR-γ expression

The regulatory role of PPAR-γ extends far beyond the transcriptional level. Epigenetic and post-translational modifications constitute a more dynamic layer of regulation, enabling cells to respond rapidly to changes in their microenvironment. This underlies the functional diversity of PPAR-γ and the seemingly contradictory phenomena associated with it.

Non-coding RNAs constitute a key upstream network regulating PPAR-γ expression (96–98) (Table 1). During the early stages of adipocyte differentiation, microRNAs such as miR-27a and miR-130 act as “brakes” by degrading PPAR-γ mRNA or inhibiting its translation, thereby preventing premature differentiation of preadipocytes (99–101). Some long non-coding RNAs regulate PPAR-γ expression through different mechanisms. For example, Snhg3 silences PPAR-γ transcription by promoting the deposition of the repressive histone modification H3K27me3 (102). In contrast, H19 lifts the suppression of PPAR-γ by competitively binding to miR-130b-3p (103). It is worth noting that there is a bidirectional regulatory relationship between PPAR-γ and these non-coding RNAs. For example, PPAR-γ can transcriptionally activate the long non-coding RNA Obr, thereby forming a positive feedback loop that promotes lipid accumulation (104). Furthermore, changes in chromatin conformation (such as circularization) can physically enhance the synergistic interaction between PPAR-γ and C/EBPα (105). Imbalances in the aforementioned regulatory network are closely associated with obesity. Under conditions of nutritional excess, the expression profiles of certain microRNAs undergo significant changes. This leads to a weakened or abnormally enhanced “inhibitory” effect on PPAR-γ. However, there is currently a lack of systematic comparisons of the relative contributions of different non-coding RNAs in different anatomical regions of adipose tissue and at different stages of obesity. This limits our in-depth understanding of their pathological significance.

**TABLE 1:** Key miRNAs and lncRNAs regulating PPAR-γ and adipose metabolism.

Category	Molecules	Primary targets/regulatory pathways	Functional effects	References
miRNA	miR-433-3p, miR-485-3p, miR-409-3p, miR-495-3p	PPAR-γ/PIK3R3	Inhibit preadipocyte differentiation	(114)
	miR-130b-3p	PPAR-γ and its downstream target genes	Inhibit adipogenesis and exert a protective effect against lipotoxicity	(24, 101, 115)
	miR-34a	PPAR-γ	Promote lipid metabolism disorders	(24, 116)
	miR-548ay-3p	PPAR-γ	Adipokine dysregulation	(117)
	miR-27a-3p	PPAR-γ/IRS1/PI3K/AKT	Promote insulin resistance	(115)
	miR-6402	BMP4/BMPR2/C/EBPβ/PPAR-γ	Reduce adipose expansion	(99)
	miR-323-5p	p-Ser112 PPAR-γ	Increase inhibitory phosphorylation and inhibit adipogenesis	(118)
	miR-6838-5p	BACE1	Improve insulin resistance and immune response	(63)
	miR-33 miR-144 miR-122	SREBP, ABCA1, PPAR-γ	Regulate lipid metabolic reprogramming such as cholesterol synthesis/efflux	(100)
lncRNA	Snhg3	Recruit the SND1 complex to catalyze H3K27me3 modification in the PPAR-γ promoter region, inhibiting its transcription	Inhibit PPAR-γ activity and drive hepatic steatosis	(102)
	H19	miR-130b-3p, miR-138/PPAR-γ	Promote abnormal adipogenic differentiation	(103, 119)
	Obr	Positive feedback loop of “PPAR-γ?Obr?lipid accumulation”	Promote lipid accumulation through epigenetic reprogramming and exacerbate the vicious cycle of obesity	(104)
	Lexis	UCP1/PPAR-γ	Enhance energy expenditure	(120)
	NR_015556	PPAR-γ/C/EBPα	Promotes adipocyte differentiation	(121)
	OIP5-AS1	PPAR-γ/AMPK/Akt/mTOR	Alleviate lipopolysaccharide (LPS) -induced inflammation	(122)
	Blnc1	PPAR-γ/SIRT6/FoxO3	Enhance inflammatory response	(123)
	MIR4435-2HG	ENO2 and PPAR-gamma	Promote lipid metabolism reprogramming	(124)
	LncRNA TUSC7	TUSC7/miR-449a/PPAR-γ and CD36 Genes	Reducing inflammation through the activity of microglia and the phagocytosis of Aβ plaques	(125)
	LINC00278	BRG1/PPAR-γ2 pathway	Promote dipsogenesis	(97)

At the post-translational modification level, lysine acetylation/deacetylation is widely regarded as the central “molecular switch” regulating PPAR-γ transcriptional activity (106, 107). Under physiological conditions, p300/CBP-mediated acetylation typically suppresses PPAR-γ activity, whereas SIRT1-mediated deacetylation restores its transcriptional function (108–111). However, the pathological significance of this modification exhibits significant context-dependence. In lipotoxic environments, activation of deacetylases is conversely associated with insulin resistance (112). In contrast, in models of liver fibrosis, PPAR-γ deacetylation exerts a protective effect (113). These contradictions suggest that the biological outcomes of acetylation are jointly influenced by tissue type, pathological context, and the site of modification. Acetylation at different PPAR-γ subtypes and sites produce distinctly different downstream effects. For example, in mammary cells, it regulates lipid synthesis rather than classical differentiation pathways (107).

Taken together, epigenetic and post-translational modifications form a multi-layered, integrated regulatory network. Non-coding RNAs and acetylation modifications synergistically shape PPAR-γ function at the levels of chromatin state and protein activity, respectively. Under physiological conditions, this network maintains a dynamic equilibrium of PPAR-γ activity, ensuring adaptive expansion of adipose tissue. However, under metabolic stress conditions such as obesity, excess nutrition and inflammatory signals disrupt this balance, leading to a functional remodeling of PPAR-γ. This drives pathological fat accumulation and insulin resistance. Future research should aim to construct dynamic network models that integrate multi-level regulatory mechanisms. This will provide a theoretical basis for precisely targeting the beneficial functions of PPAR-γ while avoiding adverse effects.

## 4 Pathological association between PPAR-γ dysfunction and obesity

### 4.1 The lineage of PPAR-γ functional states

Traditional views have simplified the role of PPAR-γ in obesity into a linear “master regulator” model (66, 68). However, a wealth of evidence reveals a central paradox: PPAR-γ can both improve systemic insulin sensitivity and lead to weight gain and adipose tissue inflammation (126–128) (Table 2). This contradiction suggests that the function of PPAR-γ is highly dependent on its activity level, the tissue microenvironment, and the specificity of downstream signaling pathways. To explain this complexity, this paper proposes a “functional continuum” model. This model conceptualizes the functional state of PPAR-γ as a continuum ranging from “underactive” to “moderately activated” to “overactivated.” Moderate activation maintains metabolic homeostasis, whereas deviation toward either extreme leads to different types of metabolic dysregulation.

**TABLE 2:** Dual effects of PPAR-γ in adipogenesis.

Type of action	Effect	Mechanism of action	References
Protective effects	Improving insulin sensitivity	Enhancing the sensitivity of fat cells to insulin, representing the core mechanism of TZDs hypoglycemic agents	(24, 62, 65, 87, 148)
	Promoting the healthy storage of fat	Inducing normal differentiation of precursor adipocytes, enhance subcutaneous fat storage capacity, direct lipids toward adipose tissue, and reduce ectopic deposition (such as in the liver), thereby improving lipid metabolism	(131)
	Inducing browning of white adipose tissue	Activating the beige/brown fat thermogenesis gene program to increase energy expenditure	(8, 110, 149–151)
	Exerting anti-inflammatory effects	Inhibiting the production of pro-inflammatory factors such as TNF-α and IL-6 in adipose tissue and macrophages, alleviating obesity-associated chronic low-grade inflammation	(90, 138–140)
	Regulate the secretion of beneficial fat factors	Increasing secretion of adiponectin ADIPOQ and leptin	(152)
	Other metabolic benefits	Improving gut-liver axis function to indirectly alleviating metabolic disorders	(22, 153)
Obesity-promoting effects	Primary regulatory factor of fat formation	It is a necessary and sufficient condition for adipocyte differentiation. Its excessive activation directly drives precursor cells to differentiate into mature adipocytes, increasing the number of fat cells and leading to obesity.	(86, 144–146)
	Promoting lipid storage	Upregulating the expression of lipogenic proteins such as fatty acid synthase, lipoprotein lipase, and fatty acid-binding proteins, thereby promoting fatty acid uptake and the synthesis and storage of triglycerides	(86, 138, 154)
	Side effects of clinical drugs	Full agonists (such as TZDs) commonly cause weight gain, fluid retention, and subcutaneous fat accumulation in clinical settings, which directly reflects their anabolic and lipogenic effects.	(86)
	Interactions with other pathways	Insulin promotes PPARγ expression, forming a positive feedback loop that may exacerbate adipocyte differentiation under hyperinsulinemic conditions.	(82, 95)

When PPAR-γ activity is impaired, preadipocyte differentiation is disrupted, leading to a reduction in subcutaneous fat storage capacity (129, 130). This forces excess lipids to be deposited in the liver, muscles, and pancreas, directly triggering insulin resistance and β-cell dysfunction (85, 131–133). A mouse model with adipose tissue-specific PPAR-γ knockout confirms this pathological process. This model exhibits severe systemic insulin resistance (134). The above evidence supports the “adipose storage capacity hypothesis.” That is, adipose tissue with normal storage function acts as a “buffer” for metabolic health, and PPAR-γ exerts a metabolic protective effect by maintaining this function (135–137). PPAR-γ safeguards adipose tissue function by preserving normal lipid storage capacity in adipocytes and preventing lipotoxicity (138). Furthermore, under conditions of PPAR-γ deficiency, inflammation in adipose tissue is alleviated through the suppression of pro-inflammatory cytokine release and the blocking of pro-inflammatory signaling pathways (139, 140).

Under physiological conditions, moderate activation of PPAR-γ maintains normal adipose tissue function. However, the quantitative criteria for what constitutes “moderate” activation remain unclear. When activity exceeds physiological thresholds and enters a state of excessive activation, functional output undergoes a qualitative change. The therapeutic paradox of TZDs provides a classic example of this. TZDs potently activate PPAR-γ, thereby improving insulin sensitivity. However, this is accompanied by side effects such as adipose tissue hyperplasia, weight gain, and edema (141–143). Mechanistically, supraphysiological activation of PPAR-γ drives excessive adipocyte hypertrophy, leading to local hypoxia, inflammation, and pathological angiogenesis, which ultimately impairs adipose tissue function (86, 144–146). This paradox raises a central question: Is it possible to decouple the beneficial metabolic effects of PPAR-γ from its pro-obesity side effects? The “selective PPAR-γ modulation” (SPPARM) strategy offers a direction for exploration. Research on “bidirectional regulatory molecules” such as Zbtb9 has provided preliminary insights (13, 20, 147). However, their molecular basis, tissue specificity, and long-term safety remain to be validated.

### 4.2 PPAR-γ dysregulation and obesity related diseases

The dangers of obesity extend far beyond adipose tissue itself, primarily due to the systemic metabolic disorders it triggers (76, 155–157). Dysfunction of PPAR-γ serves as the pivotal molecular bridge linking adipose tissue dysfunction to multi-organ pathologies involving the liver, bones, joints, and other organs (158, 159).

In the progression of non-alcoholic fatty liver disease (NAFLD), PPAR-γ exhibits a classic double-edged sword effect (160). In the liver, moderate PPAR-γ activity supports lipid metabolism and alleviates inflammation, yet its signaling network is highly susceptible to imbalance. On one hand, PPAR-γ expression and function in hepatocytes are finely regulated by the lncRNA/miRNA-138 axis, and its dysregulation exacerbates steatosis and inflammation (119). On the other hand, inactivation of protective pathways like the AMPK/PPAR-γ/Nrf2 axis renders the liver more vulnerable to oxidative stress and inflammatory assaults (161). More critically, active inter-organ communication exists between adipose tissue and the liver. Inflammatory mediators and extracellular vesicles released by dysfunctional adipose tissue remotely suppress the beneficial functions of hepatic PPAR-γ. Conversely, PPARα/γ dual agonists may disrupt this vicious cycle through systemic anti-inflammatory effects (162). This suggests that PPAR-γ-targeted therapies require an organ-specific perspective.

In obesity-associated osteoarthritis (OA), PPAR-γ serves as a key mediator for metabolic inflammation eroding joints. The classic “mechanical overload” theory no longer fully explains the complexity of obesity-related OA. Studies reveal that inflammatory mediators released by adipose tissue reach joints via the bloodstream, with disruption of the PPAR-γ/NF-κB signaling balance being a central mechanism. For instance, CDK5 accelerates joint degeneration by inhibiting PPAR-γ in macrophages and chondrocytes while activating NF-κB, thereby driving local chronic inflammation and chondrocyte apoptosis (163). This clearly delineates a metabolic-inflammatory axis “obesity/systemic low-grade inflammation/intra-articular PPAR-γ inhibition/NF-κB activation/cartilage destruction.”

The role of PPAR-γ in obesity and related diseases is a dynamic outcome determined by its activity levels, tissue-specific regulatory networks, and inter-organ crosstalk. As a central hub, it undergoes multi-level regulation from inflammatory signals, kinases, non-coding RNAs, and stability-regulating proteins. This intricate interplay dictates its ultimate pathological effects in diverse tissues such as the liver and joints. Future research should focus on mapping the functional activity profiles of PPAR-γ across different tissues and pathological stages. Therapeutic strategies must concentrate on restoring PPAR-γ equilibrium within specific tissue microenvironments, rather than simplistic activation or inhibition. Only through this approach can the therapeutic paradox be resolved, paving the way for true precision metabolic therapy.

## 5 Obesity intervention strategies by targeting PPAR-γ

### 5.1 Limitations of current drugs and directions for improvement

**FIGURE 2:**
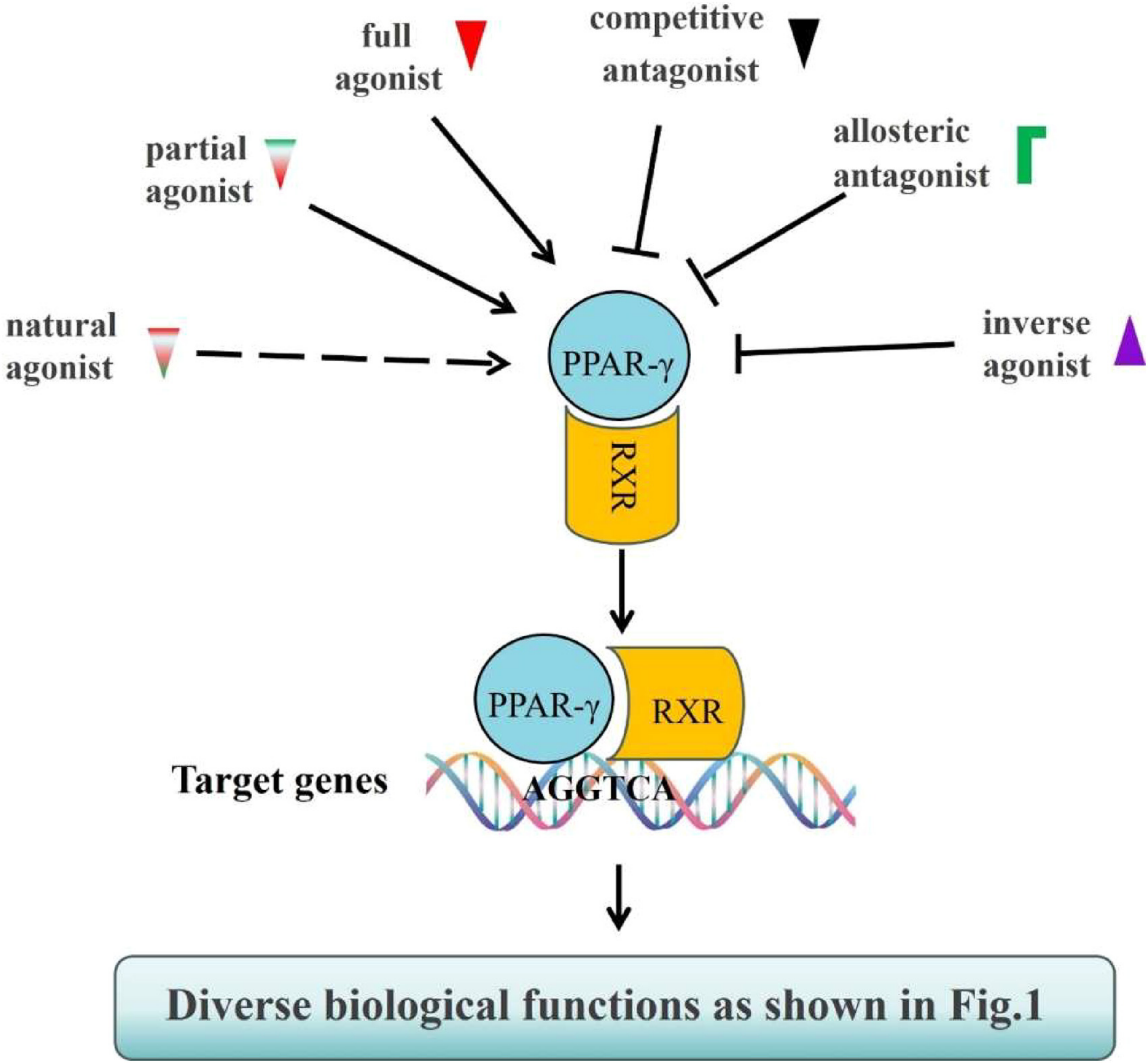
Schematic representation of drugs that affect PPAR-γ activity including agonists and antagonists. Agonists includes full agonists such as TZDs; Partial/selective agonists such as telmisartan and nateglinide metabolites; and natural agonists such as Fatty acids and 15d-PGJ2. Some PPAR-γ antagonists have been identified, including competitive antagonist such as BADGE, apigenin and luteolin; allosteric antagonists bind to regions outside the primary ligand-binding pocket, such as SR1664; inverse agonists bind PPAR-γ in a way that reduces its basal activity, such as SR-202 and SR-2595. In addition, some covalent (irreversible) Antagonists form a covalent bond with the ligand-binding pocket and permanently block agonist binding, such as GW9662 and T0070907. These drugs associate with PPAR-γ molecule in diverse pattern and affect biological function of PPAR-γ downstream as described in Figure 1.

PPAR-γ is widely recognized as a key target in obesity and metabolic diseases (34, 164). Drug development targeting PPAR-γ is shifting from the question of “whether to activate the receptor” to “how to achieve selective regulation.” Based on clinical stage, current strategies can be categorized into three groups: classic TZDs (approved), SPPARMs (clinical trials/preclinical), and tissue-specific delivery (preclinical) (Figure 2 and Table 3).

**TABLE 3:** Drugs targeting PPAR-γ and their underlying mechanisms.

Category	Representative drugs	Mechanism	Limitations	References
Dual/pan-PPAR agonist	Lanifibranor Chiglitazar	PPAR-α/δ/γ pan agonists	Weight gain, increased fracture risk, bone loss, mild gastrointestinal side effects, hemoglobin decrease	(177–179)
TZDs	Saroglitazar	PPAR-α/γ dual agonists	Weight gain, risk of heart failure	(177, 179)
	Rosiglitazone Lobeglitazone Pioglitazone	Selective PPAR-γ agonists		(165, 180, 181)
Synthetic antagonist	GW9662	Selective PPAR-γ antagonist	Preclinical research phase	(172, 173)
Inverse agonist	T0070907	PPAR-γ antagonist		(30, 182, 183)
	SR-202	Covalent PPAR-γ inverse agonist		(184)
	BAY-4931 BAY-0069			(185, 186)
	FX-909			(186, 187)

TZDs, represented by rosiglitazone and pioglitazone, are clinically approved PPAR-γ full agonists. They can significantly improve insulin sensitivity but are associated with weight gain, edema, and an increased risk of fractures (165–167). Due to their systemic, non-selective, and potent activation of PPAR-γ, these agents, while improving metabolism, also excessively stimulate fat storage and sodium-water retention pathways (32, 168, 169). Safety profiles vary among different TZDs. Rosiglitazone carries cardiovascular risks, while pioglitazone is associated with controversy regarding bladder cancer. These factors limit their widespread use in the treatment of obesity.

To overcome the limitations of TZDs, the SPPARMs strategy was developed. It aims to selectively recruit specific co-regulators by inducing conformational changes in PPAR-γ, thereby decoupling metabolic benefits from adverse effects (170, 171). Representative compounds include INT131 (discontinued after entering Phase II clinical trials), GQ-16 (preclinical), and GW9662 (preclinical/tool molecule). Preclinical studies indicate that these compounds are comparable to TZDs in improving insulin sensitivity while exhibiting reduced side effects. However, clinical translation has been hindered: INT131 was discontinued due to suboptimal efficacy, and GW9662 is used solely as a tool molecule (172, 173). This strategy remains experimental, and its molecular basis for functional selectivity and long-term safety require further validation.

Tissue-specific drug delivery represents another experimental approach. By conjugating PPAR-γ ligands with carriers that target adipose tissue, it is possible to increase local drug concentrations in fat while reducing systemic exposure. For example, preclinical models have shown that thiazolidinedione-gallate conjugates and 11β-hydroxysteroid dehydrogenase type 1 modulators improve metabolism and alleviate adipocyte hypertrophy by downregulating PPAR-γ while simultaneously activating FXR, and exhibit higher safety profiles (174–176). However, the vast majority of studies on this strategy remain in the preclinical stage.

Overall, TZDs are the only class with clinically proven efficacy. However, safety concerns regarding this class cannot be overlooked. SPPARMs and tissue-specific delivery represent promising experimental avenues, but there is a lack of confirmatory clinical data. Future research should focus on elucidating the mechanisms of functional selectivity and developing more reliable preclinical models.

### 5.2 Combined targeting of PPAR-γ and other metabolic pathways

Obesity is a systemic metabolic disorder. Monotherapy targeting PPAR-γ alone has limited efficacy. Combination therapy has emerged as an important therapeutic approach (81, 188). The combination of PPAR-γ agonists and AMPK activators (such as metformin) can synergistically improve metabolic homeostasis in multiple organs via the AMPK-PPAR-γ-endoplasmic reticulum stress axis (189). Similarly, combined targeting of PPAR-γ and the GLP-1 receptor can simultaneously improve insulin sensitivity, control appetite, and reduce body weight (190). However, combined strategies carry risks such as drug interactions, cumulative adverse effects, and weight gain that may offset weight loss. Long-term safety and optimal dosage ratios remain unclear. Future research should focus on elucidating the mechanisms of signal network interactions and validating the benefit-risk ratio through large-scale clinical trials.

### 5.3 A new perspective on PPAR-γ-mediated adipose browning

Browning of fat increases energy expenditure by converting white fat into thermogenic beige/brown fat. This represents an innovative anti-obesity strategy distinct from traditional “fat formation inhibition” approaches (111, 191–195). PPAR-γ plays a dual role in this process. It regulates lipid storage while driving the transition to a thermogenic phenotype (149–151). Based on the strength of evidence, interventions can be classified into three categories. Physiological stimuli (cold exposure, exercise) are effective in animal models but show weak effects in humans and poor compliance (196, 197); clinical interventions (famotidine, electroacupuncture) have demonstrated some browning effects (198–200), though with limited sample sizes; natural compounds, such as silymarin, enhance PPAR-γ activity via the AMPK/SIRT1/PGC-1α pathway in preclinical studies (110). However, total brown fat mass and drug responsiveness in humans are lower than in mice. Most strategies effective in rodents have not been successfully translated to the clinical setting. Furthermore, excessive activation of PPAR-γ may lead to fibrosis rather than healthy browning. Additionally, safety concerns such as compensatory increases in appetite and cardiovascular effects have not been fully evaluated. Future efforts should prioritize rigorous clinical trials and the development of precision regulatory strategies targeting thermogenic-specific nodes.

### 5.4 Gut microbiota targeting PPAR-γ and obesity

The association between the gut microbiota and its metabolites and PPAR-γ signaling offers a new perspective for obesity intervention. However, the existing evidence is primarily correlational, and a causal relationship has not yet been established (201–204). Specific strains (such as PV-1, BT-1, and LF-166) and metabolites (such as butyrate) have been shown in animal models to influence PPAR-γ activity (205). However, most studies have small sample sizes, lack independent validation, and do not employ rigorous methods for causal inference, such as germ-free animals or fecal microbiota transplantation. Probiotic interventions (e.g., Lactobacillus plantarum) are generally plagued by insufficient protocol standardization, lack of placebo controls, and poor reproducibility of results (22, 206). Furthermore, significant interindividual variations in microbial composition and metabolite absorption efficiency further complicate the challenge of achieving consistent effects. Future research should prioritize rigorous causal inference designs (such as two-way Mendelian randomization) and standardized, multicenter human studies. Until more robust mechanistic evidence is obtained, microbiome-based strategies targeting PPAR-γ should be considered experimental approaches rather than established therapeutic pathways.

### 5.5 New strategies via PPAR-γ in precision medicine

Precision medicine targeting PPAR-γ requires distinguishing between near-term feasible strategies and long-term exploratory approaches. Near-term feasible strategies include: using biomarkers (such as the Pro12Ala polymorphism and microRNA profiles) for patient stratification to identify the subgroups that will derive the greatest benefit from TZDs (65, 148, 207); developing selective inhibitors targeting specific post-translational modifications (e.g., Ser273 phosphorylation) to correct pathological functions while preserving physiological functions (208, 209); Long-term exploratory directions, such as tissue-specific CRISPR gene editing, although theoretically promising (62). However, it faces multiple barriers, including delivery efficiency, off-target effects, long-term safety, and ethical concerns. In the foreseeable future, its application will be limited to extreme case studies. Overall, biomarker-based stratification is the closest to clinical translation but requires prospective validation. Post-translational modification inhibitors are still in the preclinical stage. Meanwhile, the widespread clinical application of gene editing methods remains a distant prospect. In the future, priority should be given to promoting prospective clinical trials guided by biomarkers.

## 6 Perspective

While the central role of PPAR-γ in adipogenesis and obesity has been firmly established, numerous scientific questions remain unresolved. Addressing these gaps will not only advance mechanistic understanding of obesity pathogenesis but also inform next-generation therapeutic strategies.

Age-related functional changes in PPAR-γ. Aging is accompanied by adipose tissue remodeling, the accumulation of senescent cells, and a decline in metabolic capacity. However, it remains unclear how aging regulates PPAR-γ activity through changes in chromatin accessibility, post-translational modifications, or interactions with aging pathways. Future research should systematically map the dynamic trajectory of PPAR-γ function across the lifespan and evaluate the feasibility of stage-specific interventions during the aging process. This holds promise for developing new strategies to maintain metabolic health throughout the entire lifespan.

Gender differences in PPAR-γ function. Epidemiological studies indicate that the prevalence of obesity is higher among women. However, there are significant differences in fat distribution and metabolic risk profiles compared to men (210). Hormonal factors such as estrogen may interact with the PPAR-γ signaling pathway, driving gender-specific transcriptional responses in adipose tissue (211). Future research should prioritize large-scale, gender-stratified cohort studies. These studies should clarify gender-dependent differences in PPAR-γ-related pathways and assess whether there are systematic biases in the benefit-risk ratios of existing interventions across different genders. This will lay the foundation for designing gender-specific strategies for obesity prevention and treatment.

Optimizing the pathway from mechanism discovery to clinical translation. The core bottleneck currently facing PPAR-γ research is not a lack of targets or mechanisms, but rather the inefficiency of clinical translation. In the future, a standardized preclinical evaluation system must be established. This should include unified criteria for screening functional selective compounds, more predictive humanized animal models, and quantifiable indicators for assessing tissue-specific effects. Furthermore, priority should be given to promoting biomarker-guided prospective clinical trials to validate the practical clinical value of patient stratification strategies.

Advancing PPAR-γ research requires a focus on achieving systematic breakthroughs across three key dimensions: age, gender, and translational pathways. By addressing these knowledge gaps and optimizing the translational pathway from the laboratory to the clinical setting, substantial progress can be made in the precision intervention of obesity and related metabolic disorders.
